# Evaluating Sex and Age Differences in ADI-R and ADOS Scores in a Large European Multi-site Sample of Individuals with Autism Spectrum Disorder

**DOI:** 10.1007/s10803-018-3510-4

**Published:** 2018-02-21

**Authors:** J. Tillmann, K. Ashwood, M. Absoud, S. Bölte, F. Bonnet-Brilhault, J. K. Buitelaar, S. Calderoni, R. Calvo, R. Canal-Bedia, R. Canitano, A. De Bildt, M. Gomot, P. J. Hoekstra, A. Kaale, H. McConachie, D. G. Murphy, A. Narzisi, I. Oosterling, M. Pejovic-Milovancevic, A. M. Persico, O. Puig, H. Roeyers, N. Rommelse, R. Sacco, V. Scandurra, A. C. Stanfield, E. Zander, T. Charman

**Affiliations:** 1 Department of Psychology, Institute of Psychiatry, Psychology & Neuroscience, King’s College London, De Crespigny Park, Denmark Hill, London, SE5 8AF UK; 2 Department of Forensic and Neurodevelopmental Sciences, Institute of Psychiatry, Psychology and Neuroscience, King’s College London, De Crespigny Park, Denmark Hill, London, SE5 8AF UK; 3 Newcomen Children’s Neurosciences Centre, Evelina London Children’s Hospital at Guy’s and St Thomas’ NHS Foundation Trust, London, SE1 7EH UK; 4 Division of Neuropsychiatry, Department of Women’s and Children’s Health, Center for Neurodevelopmental Disorders (KIND), Karolinska Institutet Stockholm, Stockholm, Sweden; 5 Child and Adolescent Psychiatry, Center of Psychiatry Research, Stockholm County Council, Stockholm, Sweden; 6 UMR930, INSERM, Université François–Rabelais de Tours, Tours, France; 7 Department of Cognitive Neuroscience, Radboudumc, Donders Institute for Brain, Cognition and Behaviour, Nijmegen, The Netherlands; 8 Department of Developmental Neuroscience, IRCCS Stella Maris Foundation and University of Pisa, Pisa, Italy; 9 Department of Child and Adolescent Psychiatry and Psychology, CIBERSAM, Hospital Clínic, Barcelona, Spain; 10 Instituto Universitario de Integración en la Comunidad (INICO), Universidad de Salamanca, Salamanca, Spain; 11 University Hospital of Siena, Siena, Italy; 12 Department of Child and Adolescent Psychiatry, University of Groningen, University Medical Center Groningen, Groningen, The Netherlands; 13 Accare, Child and Adolescent Psychiatry, Groningen, The Netherlands; 14 Oslo University Hospital, Oslo, Norway; 15 Institute of Health and Society, Newcastle University, Newcastle upon Tyne, UK; 16 Sackler Institute for Translational Neurodevelopment, Institute of Psychiatry, Psychology and Neuroscience, King’s College London, De Crespigny Park, Denmark Hill, London, SE5 8AF UK; 17 Karakter Child and Adolescent Psychiatry University Centre, Nijmegen, The Netherlands; 18 School of Medicine, Institute of Mental Health, University of Belgrade, Belgrade, Serbia; 19 Interdepartmental Program “Autism 0-90”, “Gaetano Martino” University Hospital, University of Messina, Messina, Italy; 20 Mafalda Luce Center for Pervasive Developmental Disorders, Milan, Italy; 21 Department of Experimental-Clinical and Health Psychology, Ghent University, Ghent, Belgium; 22 Service for Neurodevelopmental Disorders, University Campus Bio-Medico, Rome, Italy; 23 University of Edinburgh, Edinburgh, UK; 24 Department of Clinical Sciences, Child and Adolescent Psychiatry, Medical Faculty, Lund University, Lund, Sweden; 25 Child and Adolescent Psychiatry Helsingborg, Psychiatry Skåne, Region Skåne, Sweden; 26 Department of Women and Children’s Health, School of Life Course Sciences, Faculty of Life Sciences and Medicine, King’s College London, London, UK; 27 Department of Special Needs Education, University of Oslo, Oslo, Norway

**Keywords:** Autism Spectrum Disorder, Phenotype, Sex, Age, Symptom severity

## Abstract

Research on sex-related differences in Autism Spectrum Disorder (ASD) has been impeded by small samples. We pooled 28 datasets from 18 sites across nine European countries to examine sex differences in the ASD phenotype on the ADI-R (376 females, 1763 males) and ADOS (233 females, 1187 males). On the ADI-R, early childhood restricted and repetitive behaviours were lower in females than males, alongside comparable levels of social interaction and communication difficulties in females and males. Current ADI-R and ADOS scores showed no sex differences for ASD severity. There were lower socio-communicative symptoms in older compared to younger individuals. This large European ASD sample adds to the literature on sex and age variations of ASD symptomatology.

**Electronic supplementary material:**

The online version of this article (10.1007/s10803-018-3510-4) contains supplementary material, which is available to authorized users.

## Introduction

Autism Spectrum Disorder (ASD) is one of the most common neurodevelopmental conditions with a prevalence of 1–1.5% of children and adults (Baird et al. 2006; Brugha et al. 2011; Christensen et al. 2016). A consistent finding from both clinical observation and empirical evidence is that more males than females are diagnosed with ASD, and current estimates range from 3:1–4.3:1 across the autism spectrum (Loomes et al. 2017). This ratio, however, varies as a function of IQ, with prevalence rates of 5.75:1 males: females in samples composed of individuals in the normative IQ range (> 70) compared to 1.9:1 in ASD associated with low IQ (≤ 70) (Baird et al. 2006; Fombonne 2009; Scott et al. 2002; Kim et al. 2011). The reason for this discrepancy in the sex ratio is unclear. While some have suggested that females may require a greater genetic load to develop ASD (Jacquemont et al. 2014), others have proposed that the male-preponderance in ASD prevalence, particularly at the intellectually able end of the spectrum, may be related to females being better at compensating for their difficulties (“female camouflage”); (Attwood 2006; Lai et al. 2011; Postorino et al. 2015; Rynkiewicz et al. 2016), potentially leading to under-recognition of females and delay in diagnosis (Lai et al. 2015). Indeed, there is evidence from population studies that girls with comparable levels of symptoms to boys are less likely to be diagnosed or are later diagnosed by community services (Russell et al. 2011; Kirkovski et al. 2013), unless they present with more substantial behavioural and/or cognitive difficulties (Dworzynski et al. 2012).

The way the core clinical symptoms of ASD—difficulties in social communication and interaction and the presence of restricted, repetitive, behaviours and interests and atypical responses to sensory input (DSM-5, American Psychiatric Association 2013)—manifest may also be different for males and females (Mandy et al. 2012; Van Wijngaarden-Cremers et al. 2014). Yet, in contrast to the strong evidence of sex differences in the prevalence of ASD, differences between the sexes in the phenotypic presentation of ASD have been found to be small in magnitude and available findings are inconsistent, both in terms of the severity of core symptoms and across age and level of functioning. While some studies have found no significant sex differences in the behavioural presentation of ASD using the ADOS (Lord et al. 2000, 2012; Ratto et al. 2017) and/or ADI-R (Rutter et al. 2003; Holtmann et al. 2007; Pilowsky et al. 1998; Andersson et al. 2013; Reinhardt et al. 2015; Harrop et al. 2015; Ratto et al. 2017), others have reported some differences using a mixed set of measures (for reviews see Lai et al. 2015; Kirkovski et al. 2013; Van Wijngaarden-Cremers et al. 2014). For example, a meta-analysis of smaller-scale studies (Van Wijngaarden-Cremers et al. 2014) and multi-site large-scale studies (Mandy et al. 2012; Szatmari et al. 2012; Frazier et al. 2014; Supekar and Menon 2015; Charman et al. 2017) demonstrated fewer restrictive and repetitive behaviours (RRB) in females than males, consistent with findings both in young children with varying cognitive abilities (Lord et al. 1982; Hartley and Sikora 2009) and intellectually able adults (Wilson et al. 2016; Lai et al. 2011). In contrast, specific sex differences in the severity of social and communication impairments have not been conclusively presented. Some studies have found girls to have more impaired social and/or communicative functioning than boys (Hartley and Sikora 2009; Carter et al. 2007), whereas others have found equivalent (Wilson et al. 2016; Mandy et al. 2012; Supekar and Menon 2015) or superior social and communication skills in females compared to males (Lai et al. 2011; Park et al. 2012). Comparisons between studies are compromised by a number of factors that potentially contribute to the discrepancy in findings.

First, females with ASD are often underrepresented due to small sample sizes that result in limited statistical power to detect small to moderate effects. Studies involving intellectually able adolescents and adults are particularly affected by this problem, and while some have addressed this issue by analysing large-scale datasets (Mandy et al. 2012; Frazier et al. 2014; Howe et al. 2015; Supekar and Menon 2015; Wilson et al. 2016; Charman et al. 2017), these studies have been limited. Second, although the ASD phenotype may present differently in males and females, current defining (DSM) criteria are still mainly based on male characteristics. This is true from both a qualitative and a quantitative point of view, because diagnostic thresholds are similar in males and females (Tsai and Beisler 1983; McLennan et al. 1993; Holtmann et al. 2007; Lai et al. 2015). This poses several problems. If current diagnostic criteria are more tuned to the male phenotype of ASD, the diagnosis of ASD in females may be missed or the condition could be misdiagnosed (Rivet and Matson 2011; Begeer et al. 2013; Dworzynski et al. 2012), even if these females present with a substantial clinical burden and would benefit from support programmes. Moreover, since sex differences in presentation may not lead to a diagnosis in females, many ASD samples potentially miss a large number of females resulting in an overrepresentation of males in ASD research even if a small group of females is included (but underrepresented) who fulfil DSM criteria, although results are thought to be applicable to both sexes (Lai et al. 2015).

Third, there is evidence that ASD symptoms may present differently across development. Some studies highlight reduced ASD symptoms with age, particularly in early childhood, but also marked heterogeneity in the trajectory of symptom expression over childhood and into early adolescence with some individuals having relatively stable high or low symptom levels across age, while others improve or become more impaired over time (Bölte and Poustka 2000; Szatmari et al. 2009, 2015; Fountain et al. 2012; Gotham et al. 2012; Lombardo et al. 2015; Bal et al. 2015). Core symptoms also often persist into adulthood, but often improve compared to adolescence (Billstedt et al. 2007; Shattuck et al. 2007; Howlin et al. 2013). Thus, comparing samples of young children (Hartley and Sikora 2009; Carter et al. 2007) to subjects across a broad age range (Pilowsky et al. 1998) may mask sex differences due to developmental changes.

Fourth, differences between males and females in the behavioural presentation of ASD may also vary with IQ, and whilst some studies have matched for IQ and age, others have not. Finally, previous studies have differed in the choice of measures used, from structured caregiver interviews (ADI-R), clinician rated observational measures (ADOS), to parent- or self-reported questionnaires, and this may have contributed to the discrepant findings (Lemler 2012; Grantham et al. 2011). The ADI-R for example probes about an individual’s current or past behaviour (ever and at 4-to-5-years—considered historically to be the ‘prototypic age’ of presentation), while the ADOS measures current symptom severity in a standardised behaviour sampling context. These instruments are relevant in our clinical and conceptual understanding of ASD symptomatology, but may yield different insights into the ASD phenotype based on their relative strengths and weaknesses in assessing symptom presentation at different developmental time-points using different informant and context-dependent assessment techniques (Charman and Gotham 2013).

Given these confounds, the pattern of sex differences in the core symptomatology of ASD remains unclear, potentially contributing to a male-bias in our understanding of ASD (for a recent special issue on this topic see Mandy and Lai 2017). One potential avenue to advance our understanding is to obtain large-scale samples which are difficult to acquire from one site alone. While some efforts are underway to actively pool clinical data from multiple sites for informative analysis (Simons Simplex Collection, Frazier et al. 2014), similar large-scale collaborative efforts have so far been largely neglected in Europe (but see Bildt et al. 2015). In response, we set up a collaboration to collect historical clinical data from ASD clinical and research institutions across Europe that are part of the EU-AIMS Clinical Network (https://www.eu-aims.eu/clinical-network/) to examine differences across the ASD phenotype according to sex and age including larger sample sizes of females with ASD than previously examined. This circumvents the previous limited size of populations studied, narrow age ranges, level of abilities and ascertainment differences. While our primary aim was to investigate sex differences in ASD symptomatology, the size of this cross-sectional dataset and broad age distribution also afforded to analyse differences in symptomatology relating to age.

## Method

### Participants

Sites in the EU-AIMS clinical network (100 sites in 37 countries; http://www.eu-aims.eu/clinical-network/) were contacted between 2015 and 2017 to indicate their willingness to share behavioural and cognitive data for secondary analysis. Of these, 18 sites from nine European countries contributed 28 datasets relevant for this study resulting in a total sample of 2684 individuals with ASD (see Table 1 for a summary of datasets by site).

**Table 1:** Consortium sites, contributors and ASD sample size for all datasets

Letter	Contributing sites (Country)	Principal investigators and key contributors	Males *n* ^*a*^	Females *n* ^*a*^	Total *N*
a	University of Oslo (Norway)	Anett Kaale	20	3	23
b	Evelina London Children’s Hospital—Guy’s and St Thomas (UK)	Michael Absoud	23 (2)	2	25
c	Ghent University (Belgium)	Herbert Roeyers	18 (2)	7 (3)	25
d	University of Edinburgh (UK)	Andrew Stanfield	32	13	45
e	Hospital Clinic of Barcelona (Spain)	Rosa Calvo	45	3	48
f	University Hospital of Siena (Italy)	Roberto Canitano	47	7	54
g	Newcastle University (UK)	Helen McConachie	61	9	70
h	University of Tours (France)	Frédérique Bonnet-Brilhault	64	10	74
i	Karakter (The Netherlands)	Iris Oosterling/Jan Buitelaar	126	33	159
j	University Campus Bio-Medico (Italy)	Antonio Persico/Roberto Sacco	168 (74)	36 (16)	204
k	IRCCS Fondazione Stella Maris (Italy)	Sara Calderoni/Antonio Narzisi	177	31	208
l	Karolinska Institutet/KIND (Sweden)	Sven Bölte/Eric Zander	161 (19)	47 (7)	208
m	University of Salamanca (Spain)	Ricardo Canal Bedia	183 (4)	30 (1)	213
n	King’s College London (UK)	Tony Charman	194	19	213
o	RadboudUMC/Karakter (The Netherlands)	Nanda Rommelse/Jan Buitelaar	176	45	221
p	Institute of Mental Health (Serbia)	Milica Pejovic-Milovancevic	202 (15)	45 (4)	247
q	King’s College London (UK)	Declan Murphy	206	68	274
r	UMC Groningen and Accare University Center (The Netherlands)	Annelies de Bildt/Pieter Hoekstra	317 (116)	56 (29)	373

Each contributing site and sample is assigned an alphabetical letter

^a^Number in brackets indicates the number of males and females with ASD and Intellectual Disability (ID) for each site

Datasets from all participating sites were obtained from a range of existing research programmes (e.g. early screening studies, intervention programs, high-risk sibling studies, genetic and imaging studies) and ascertained from a variety of settings including volunteer databases and research cohorts, clinical referrals from local outpatient centres, special needs schools, mainstream schools and local communities. Resembling DSM-5 (American Psychiatric Association 2013), diagnostic classifications used in older systems (DSM-IV/-TR, ICD-10; American Psychiatric Association 1994, 2000; World Health Organization 1992), i.e. autistic disorder, Asperger’s syndrome, atypical autism versus non-ASD were collapsed into ASD versus non-ASD. Clinical diagnosis of ASD was made according to DSM-IV (American Psychiatric Association 2000), DSM-IV-TR (American Psychiatric Association 2000), DSM-5 (American Psychiatric Association 2013) or ICD-10 criteria (World Health Organization 1992). Minimal requirements for inclusion of datasets in the study were data on the Autism Diagnostic Interview-Revised (ADI-R; summary or item-level data) and/or data on the Autism Diagnostic Observation Schedule (ADOS; item-level data), as well as basic demographic information (e.g. age, sex). To allow comparability of data across sites, data processing, coding and submission was standardised across sites by developing a common data sharing protocol and a data dictionary. Upon receipt, data were checked for impossible data entries (for example data points beyond published maxima and minima) and missing values. When item-level data was available (45% for ADI-R, 100% for ADOS), ADI-R standard algorithm scores for reciprocal social interaction (Social), communication, and restricted, repetitive and stereotyped behaviours and interests (RRB) and ADOS comparison or Calibrated Severity Scores (CSS) total, social affect (SA) and restricted and repetitive behaviours (RRB) were recomputed from the original item scores. There were no formal exclusion criteria of individuals (e.g. presence of any DSM-5 axis I and II psychiatric disorders). Institutional Review Board’s approval from King’s College London (ethics reference number: PNM/13/14-174) was obtained to collect fully anonymised data for secondary analysis to ensure confidentiality of the shared data.

### Measures

The *Autism Diagnostic Observation Schedule* (ADOS-G, Lord et al. 2000, 2012; ADOS-2) is a semi-structured observational assessment designed to evaluate aspects of communication, social interaction, play, and stereotyped behaviours and restricted interests. Depending on an individual’s language level and age, certified staff in ADOS administration (e.g. clinicians, psychologists, research staff) administered to participants one of several modules (modes of implementation) of the ADOS (see Tables 2, 3 for a summary of participants by module). The majority of individuals received Module 1 for preverbal children who use no expressive language (*N* = 484) or only single words (*N* = 374). The other modules that were administered included Module 2 for children with phrase speech (*N* = 199), Module 3 for more verbally fluent and older children (*N* = 275), as well as Module 4 for adolescents and adults with fluent speech (*N* = 88). Module T from the ADOS-2 was not represented. Across sites, the majority of individuals received the ADOS-G (*N* = 1383), while some received the ADOS-2 (*n* = 37, Stockholm site). To allow comparability across ADOS Modules, ADOS-G raw scores were mapped onto ADOS-2 raw scores and CSS were computed (Gotham et al. 2009; Hus et al. 2014). CSS provide standardised ASD severity measures across the different modules for the core symptom domains of social communication (i.e. social affect, SA) and RRB, as well as an overall indicator of ASD severity (CSS Total). This metric has been shown to be less strongly associated with age and language compared to raw ADOS-2 totals. CSS can range from 1 to 10, with higher scores indicating more severe ASD symptoms. Note that since the raw RRB total consists of only four items, the CSS-RRB encompasses a more limited range of values (i.e. 1 and 5–10).

**Table 2:** Participant characteristics overall and split by ADOS and ADI-R datasets

Statistic	All datasets	ADOS						ADI-R		
		All ADOS datasets	Module 1 No words	Module 1 Some words	Module 2	Module 3	Module 4	All ADI-R datasets	4–5 ever/historical	Current
*N* (sites)	18	11	8	10	9	7	5	16	16	7
*N* (sample)	2684	1420	484	374	199	275	88	2139	2139	1030
*N* (females)	464 (17%)	233 (16%)	91 (19%)	55 (15%)	31 (16%)	33 (12%)	23 (26%)	376 (18%)	376 (18%)	169 (16%)
Mean age (*SD*)	10.3 (9.1)	7.4 (7.5)	3.7 (3.0)	4.5 (3.0)	6.7 (3.7)	11.6 (2.4)	27.9 (13.7)	11.2 (8.9)	11.2 (8.9)	9.3 (5.8)
Age range (in years)	1–65	1–65	1–20^a^	2–20^b^	2–19^c^	3–20^d^	8–65	2–61	2–61	2–61

^a^*N* = 1 younger than 2 years and *N* = 12 individuals with intellectual disability older than 14 years were given Module 1—no words

^b^*N* = 9 individuals with intellectual disability older than 14 years were given Module 1—some words

^c^*N* = 5 individuals older than 16 years were given Module 2

^d^*N* = 11 individuals older than 16 years were given Module 3

**Table 3:** Summary of variation between datasets in demographic, behavioural characteristics and level of ASD symptomatology (split by ADOS and ADI-R datasets)

	Ranges across datasets				Variance				*x*^2^ sig. value
	Minimum	Maximum	Mean	*SD*	Overall mean (*SD*)	Within datasets	Between datasets	*ICC* ^d^	
Chronological age [years:months]	1:0–23:7^a^	2:5–65:3	2:0–39:5	0:3–11:8	7:4 (7:5)	98.6	14.84	.87	*p* < .0001
	1:5–25:8^b^	3:8–60:9	2:7–40:8	0:2–9:7	11:2 (8:9)	69.29	23.67	.75	*p* < .0001
	2:0–25:8^c^	6:1–60:9	4:3–40:8	0:7–9:7	10:7 (5:5)	99.58	14.92	.87	*p* < .0001
Sex, % of male participants			58.8–100^a^		83.6 (3.7)	0.38	0.12	.01	*p* = .017
			71.4–100^b^		82.4 (3.8)	0.37	0.04	.01	*p* = .004
			70.0–100^c^		83.9 (3.7)	0.38	0.04	.05	*p* = .0002
Nonverbal IQ	25–73^a^	92–148	54–115	11–27	74 (26)	294.28	490.83	.38	*p* < .0001
	25–75^b^	99–154	55–109	14–32	80 (27)	222.53	478.24	.32	*p* < .0001
	25–71^c^	108–154	69–109		81 (28)	257.56	513.55	.33	*p* < .0001
ADOS—CSS									
Total	1–4	8–10^e^	3–7	1–3	6 (2)	0.62	4.79	.11	*p* < .0001
SA	1–6	9–10^e^	4–8	1–3	7 (2)	0.38	4.85	.07	*p* < .0001
RRB	1–5	6–10^e^	4–7	0–3	6 (2)	0.81	5.73	.12	*p* < .0001
ADI-R—ever/diagnostic									
Social interaction	0–10	21–30	9–22	4–7	17 (7)	10.57	35.18	.23	*p* < .0001
Communication	0–7	15–27	9–14	3–5	12 (5)	3.43	20.15	.15	*p* < .0001
RRB	0–2	7–18	1–9	1–4	5 (3)	2.28	7.15	.24	*p* < .0001
ADI-R—current									
Social interaction	0–2	13–30	5–17	4–8	12 (6)	10.24	30.60	.25	*p* < .0001
Communication	0–4	13–24	5–13	3–5	9 (4)	1.40	15.54	.08	*p* < .0001
RRB	0–1	8–12	3–5	2–3	4 (2)	0.65	5.43	.11	*p* < .0001
Sample size	4^a^	199	74	62					
	8^b^	274	98	78					
	8^c^	220	96	77					

*ICC* intraclass correlation coefficient, *ADI*—*R* Autism Diagnostic Interview—Revised, *ADOS CSS Total, SA, RRB* Autism Diagnostic Observation Schedule Calibrated Severity Scores for total, social affect and restricted and repetitive behaviours, *IQ* intelligence quotient

^a^Indicators in row relate to ADOS datasets only

^b^Indicators in row relate to ADI-R 4–5 ever/diagnostic datasets only

^c^Indicators in row relate to ADI-R 4–5 current datasets only

^d^The ratio of between-dataset variance to total variance

^e^The highest possible score (i.e. ceiling) on the instrument

The *Autism Diagnostic Interview—Revised* (ADI-R, Rutter et al. 2003) was completed with parents or careers of individuals with ASD. The ADI-R is a standardised structured interview based on ICD-10 and DSM-IV diagnostic concepts of ASD and explores across 93 items an individual’s early development, language acquisition and/or loss of language, functioning of language and communication, social development and play as well as interests and behaviours, general behaviour and behavioural concerns. The interview focuses on three behavioural domains (i.e., reciprocal social interactions, language/communication, and restricted, repetitive, and stereotyped behaviours and interests), for which standard algorithm scores are derived to compute current (where available) and/or historical (4-to-5-years/ever algorithm scores) symptom scores (Table 3).

### General Intellectual Ability

Across datasets, the general level of intellectual abilities was assessed using a range of different developmentally-appropriate scales and instruments. The majority of individuals were either administered the *Wechsler Intelligence Scale for Children-III*/*IV* (WISC-III/IV; Wechsler 1991, 2003) designed for children aged 6–16 years, the *Wechsler Preschool and Primary Scale of Intelligence for Children-III*/*IV* (WPPSI-III/IV; Wechsler 2002, 2012) intended for children aged 4–6 1/2 years or the *Wechsler Adult Intelligence Scale for Adults-III*/*IV* (WAIS-III/IV; Wechsler 1997, 2008). Some adults were also assessed using the *Wechsler Abbreviated Scale of Intelligence* (WASI; Wechsler 1995). Other measures included the *Griffiths Mental Development Scales - Extended Revised* for children aged 2–8 years (GMDS-ER 2–8; Luiz et al. 2006) and the *Leiter International Performance Scale—Revised* (Leiter–R; Roid and Miller 2011) for individuals aged 2–20 years. For each measure, estimates of standard nonverbal IQ scores (NVIQ) were derived from the appropriate subtests and index scores with exception of the B-L-R, where NVIQ were derived from mean age equivalent scores of all non-verbal subscales divided by the chronological age in months * 100. This was done to maximise IQ data availability across sites.

Infants and toddlers (intended for use from age 0–69 months) received either the *Brunet-Lézine Revised* (B-L-R, Brunet et al. 1997), the *Mullen Scales of Early Learning* (MSEL; Mullen 1995), the *Merrill-Palmer-Revised* (M-P-R; Roid and Sampers 2004) or the PEP-R (Schopler et al. 1990). For the MSEL, NVIQ were derived from age equivalent scores on the on fine motor (FM) and visual reception (VR) subscale: NVIQ= (mean age equivalent on FM and VR/chronological age in months) * 100. NVIQ on the Merrill-Palmer was calculated as (mean age equivalent on cognitive and fine motor/chronological age in months) * 100, while for the PEP-R NVIQ was based on (mean developmental age in months on all subscales except for the verbal scale/chronological age in months) * 100. IQ scores lower than 20 (*n* = 26) were discarded due to difficulties in establishing a reliable IQ estimate in profound intellectual disability.

### Statistical Analysis

Linear mixed-effects models were fit using a maximum likelihood estimation method and were executed using STATA software 15.0 (StataCorp 2017). To take into consideration the multi-level nature of the data, as well as to account for heterogeneity across datasets in outcome measures, a random effect for dataset was included in all models. This affords to estimate differences between datasets in the specific populations enrolled, the differing IQ tests used, and other factors that may increase variability due to pooling individual-level data from many sources. Intraclass correlation coefficients (ICCs) reflecting the ratio of between-dataset variance to total variance are reported to provide an estimate of the amount of shared variance among individuals from the same dataset that is due to the higher-level unit only (i.e. belonging to the same dataset; see Table 3). The linear mixed-effects models yield Chi square coefficients and *p* value for categorical predictor variables (i.e. sex) and standard errors, *t*-statistics and confidence intervals for slope coefficients of continuous variables (i.e. chronological age in years, non-verbal IQ scores). To account for multiple comparisons for analyses in each measure, Bonferroni corrections were applied (corrected α-level: *p* < .016).

Analyses are reported with/without NVIQ as a continuous predictor (Tables 4, 5, respectively) to (1) capitalise on the full sample size and (2) test these effects in a sub-sample of individuals where NVIQ data was available. ADI-R 4-to-5/ever scores were analysed using a fixed effect for sex, while ADI-R current scores and ADOS CSS included fixed effects for sex and chronological age. For categorical predictors, effect sizes were calculated according to Tymms (2004) by dividing the difference in marginal means by the square root of the variance at the within-subject level. This measure of effect size is equivalent to Cohen’s d or standardised difference (Cohen 1992), where an effect size of 0.20–0.30 is taken to be a small effect, 0.50 a medium effect and greater than 0.80 a large effect. Prior to analysis, ADOS RRB CSS and both 4–5 ever/diagnostic and current scores on the ADI-R RRB domain were log-transformed to meet normality assumptions.

**Table 4:** Predicted effect of age and sex on ASD diagnostic measures using the whole sample of participants

Variable	Chronological age				Sex			Sex by age interaction			
	*b SE*(*b*)	*t*	*p* value	95% CI	*x* ^2^	*p* value	d	*b SE*(*b*)	*t*	*p* value	95% CI
ADI-R—4–5 ever/item scores^a^											
Social					0.67	0.412	0.05				
Communication					3.20	0.074	0.12				
Restricted and repetitive behaviours^b^					11.8	0.0006	0.21				
ADI-R—current item scores^c^											
Social	− 0.41 (0.04)	9.89	> 0.001	[-.52, − 0.35]	1.12	0.289	0.05	0.01 (0.08)	0.04	0.967	[-.17, 0.18]
Communication	− 0.23 (0.03)	7.63	> 0.001	[-.29, − 0.17]	0.01	0.994	0.08	− 0.03 (0.05)	0.68	0.498	[-.13, 0.06]
Restricted and repetitive behaviours^b^	0.01 (0.01)	1.62	0.105	[-.01, 0.01]	0.05	0.831	0.17	− 0.01 (0.01)	1.11	0.266	[-.03, 0.01]
ADOS Calibrated Severity Scores^d^											
Total	− 0.04 (0.01)	3.03	0.002	[-.06, − 0.01]	0.07	0.789	0.06	− 0.03 (0.02)	1.27	0.204	[-.06, 0.01]
Social affect	− 0.03 (0.01)	2.21	0.027	[-.05, − 0.01]	0.06	0.801	0.03	− 0.02 (0.02)	0.81	0.415	[-.06, 0.02]
Restricted and repetitive behaviours^b^	− 0.01 (0.01)	1.31	0.189	[-.01, 0.01]	0.22	0.643	0.17	− 0.02 (0.01)	2.90	0.004	[-.03, − 0.01]

*b* Regression coefficient, *SE*(*b*) standard error of regression coefficient, *t* t-statistic, *95% CI* 95% confidence Interval of regression coefficient, *ADI-R* Autism Diagnostic Interview—Revised, *ADOS* Autism Diagnostic Observation Schedule

^a^ADI-R 4-to-5 diagnostic/ever scores analyses: *N* = 2139 participants included

^b^Log-transformed scores

^c^ADI-R current scores analyses: *N* = 1030 participants included

^d^ADOS analyses: *N* = 1420 participants included

**Table 5:** Predicted effect of age, sex and IQ on ASD diagnostic measures in a sub-sample of participants with NVIQ scores

Variable	Chronological age				Intellectual functioning				Sex			Sex by age interaction			
	*b SE*(*b*)	*t*	*p* value	95% CI	*b SE*(*b*)	*t*	*p* value	95% CI	*x* ^2^	*p* value	d	*b SE*(*b*)	*t*	*p* value	95% CI
ADI-R—4–5 ever/item scores^a^															
Social					− .06 (.01)	8.22	< .001	[− .08, − .05]	0.92	.337	.08				
Communication					− .02 (.01)	3.17	.002	[− .03, − .01]	0.97	.334	.07				
Restricted and repetitive behaviours^b^					− .01 (.01)	3.49	< .001	[− .01, − .01]	5.07	.024	.21				
ADI-R—current item scores^c^															
Social	− .29 (.05)	5.58	< .001	[-.38, − .19]	− .06 (.01)	7.21	< .001	[− .08, − .04]	2.44	.119	.03	− .13 (.07)	1.80	.071	[− .27, .01]
Communication	− .19 (.04)	5.13	< .001	[− .26, − .12]	− .02 (.01)	3.83	< .001	[− .04, − .01]	0.49	.483	.05	− .07 (.06)	1.26	.206	[− .18, .04]
Restricted and Repetitive Behaviours^b^	− .01 (.01)	0.02	.981	[− .01, .01]	− .01 (.01)	4.13	< .001	[− .01, − .01]	1.72	.189	.30	− .01 (.01)	0.34	.737	[− .02, .02]
ADOS Calibrated Severity Scores^d^															
Total	.01 (.02)	0.12	.901	[− .03, .03]	− .03 (.01)	10.2	< .001	[− .04, − .03]	0.62	.430	.19	− .02 (.02)	1.02	.308	[− .07, .02]
Social affect	.01 (.01)	0.70	.486	[− .02, .04]	− .03 (.01)	9.45	< .001	[− .04, − .02]	1.09	.296	.17	− .01 (.02)	0.39	.694	[− .05, .04]
Restricted and repetitive behaviours^b^	.01 (.01)	0.72	.470	[− .01, .01]	− .01 (.01)	8.33	< .001	[− .01, − .01]	0.11	.739	.19	− .02 (.01)	2.74	.008	[− .03, − .01]

*b*  regression coefficient, *SE(b)*  standard error of regression coefficient, *t*  t-statistic, 95% *CI* 95% Confidence Interval of regression coefficient

*ADI-R* Autism Diagnostic Interview—Revised,* ADOS* Autism Diagnostic Observation Schedule

^a^ ADI-R 4-to-5 diagnostic/ever scores analyses: *N* = 1114 participants included

^b^ log-transformed scores

^c^ ADI-R current scores analyses: *N* = 705 participants included

^d^ADOS analyses: *N* = 846 participants included

## Results

### Sample Composition

Eighteen sites contributed 28 previously collected datasets on a total of 2,684 individuals, with contributions per site ranging from 23 to 373 participants (see Table 1). Data on the ADI-R was available for 2139 individuals (80% of the total sample), while data on the ADOS was available for 1,420 individuals (53% of the total sample). On 1030 individuals (38% of the total sample), both ADI-R and ADOS data was available—a separate analysis including only those individuals can be found in the supplementary materials. Given the limited number of individuals with both ADI-R and ADOS data, demographic information is reported for all datasets and for ADOS/ADI-R datasets separately (Table 2).

In the total sample, the mean chronological age was 10.3 (*SD* = 9.1) years, with males being on average slightly, but not significantly, younger than females overall (*M*_Male_ = 10.1, *SD*_Male_ = 9.0; *M*_Female_ = 11.2, *SD*_Female_ = 9.5, *x*^*2*^(1) = 1.05, *p* = .306, *d* = .03). The mean level of non-verbal intellectual abilities (NVIQ) was 80.9 (*SD* = 27.3; interquartile range (*IQR*) = 38), ranged from 25 to 154 and was available for 1283 subjects (ADOS datasets: *N* = 846, 60%, ADI-R diagnostic datasets: *N* = 1114, 52%; ADI-R current datasets: *N* = 705, 68%). NVIQ scores were on average significantly higher for males compared to females overall (*M*_Male_ = 81.9, *SD*_Male_ = 27.1; *M*_Female_ = 76.1, *SD*_Female_ = 27.91, *x*^*2*^(1) = 19.56, *p* < .0001, *d* = .33). Separate analyses for ADOS/ADI-R diagnostic/current datasets-only can be found in the Supplementary Materials.

**Fig. 1:**
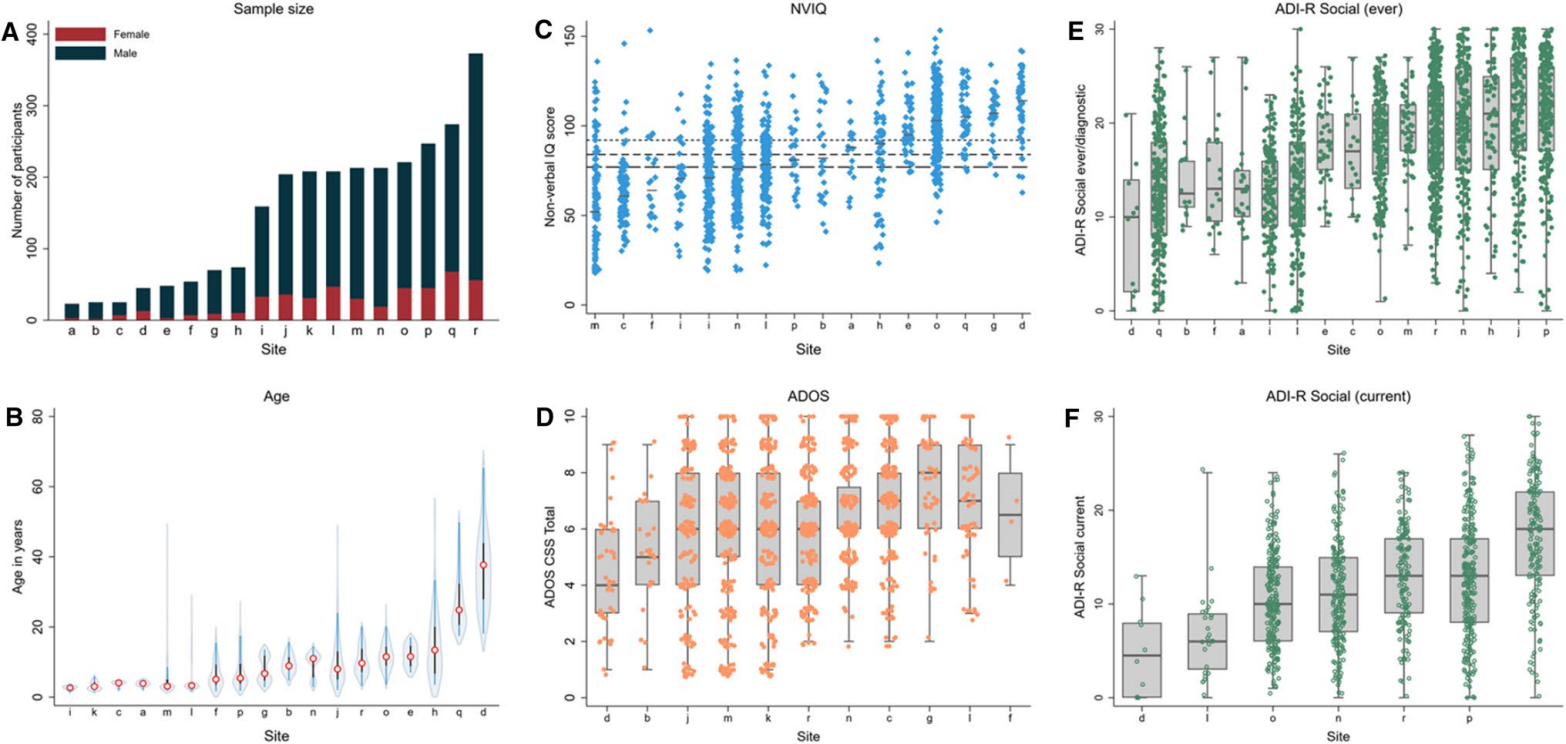
Data pooling sample characteristics. **a** Total number of participants with ASD by sex for each contributing site ordered as a function of sample size (labelled alphabetically, see Table 1 for label key). The same site labels are used for (**b**–**f**). **b**–**f** are ordered by median sample statistic per site. **b** Violin plot of chronological age in years for all individuals per site. **c** Distribution of nonverbal IQ scores per site. Short-dashed line NVIQ for ADI-R datasets, long-dashed line NVIQ for ADOS datasets. Solid black lines indicate median NVIQ per site. **d**–**f** Tukey’s box-whiskers plots overlaid with scatterplots of individual data points per site for (**d**) ADOS Calibrated Severity Scores (CSS) Total, **e** ADI-R Social scores (ever/diagnostic) and **f** ADI-R Social scores (current)

Marked variation in age and NVIQ across datasets (and for ADOS and ADI-R datasets separately) was evident alongside a large predominance of male subjects (Table 3). This is also reflected in the significant random effect for dataset included in all models for most of the key demographic and diagnostic measures. The Intra Correlation Coefficients (ICCs) indicate that whilst the effect of dataset was large for age (75–87%), reflecting the variable recruitment pattern across sites, it was moderate for NVIQ (32–38%) and 1–5% for sex ratio. On the diagnostic measures, ICCs were generally low to moderate between 7 and 12% for ADOS scores and between 8 and 25% for ADI-R scores. Figure 1 highlights the variation between sites by pooling demographic and clinical information across datasets within a site.

### ASD Measures—Effects of Sex and Age

Excluding NVIQ as a predictor in the model and using the whole sample, sex-related analyses revealed that ADI-R 4–5 diagnostic/ever scores (Total *N* = 2139) were higher in males compared to females on the RRB domain (*M*_Male_ = 5.05; *SD*_Male_ = 3.2, *M*_Female_ = 4.38; *SD*_Female_ = 3.3, *x*^*2*^(1) = 11.80, *p* = .0006, *d* = .21; see Table 4), but not on the ADI-R social domain (summary statistics can be found in Supplementary Table 1). A non-significant trend towards higher scores in males was found on the ADI-R Communication domain (*p* = .074, *d* = .12). No main effect of sex for ADOS CSS Total, ADOS SA, ADOS RRB (Total N = 1,420, all *p* > .60) and ADI-R current domain Social, Communication and RRB scores (Total *N* = 1,030, all *p* > .20) were observed. For ADOS CSS RRB, there was a significant sex by age interaction (*b* = − .02, *p* = .004), with females but not males showing significantly lower scores with increasing age. However, when restricting the analysis to individuals aged 25 or less (retaining 97% of the initial sample), the sex by age interaction was not significant (*b* = − .01, *p* = .22), suggesting that these results are likely to be driven by a small number of older adult male participants with high RRB symptoms.

**Fig. 2:**
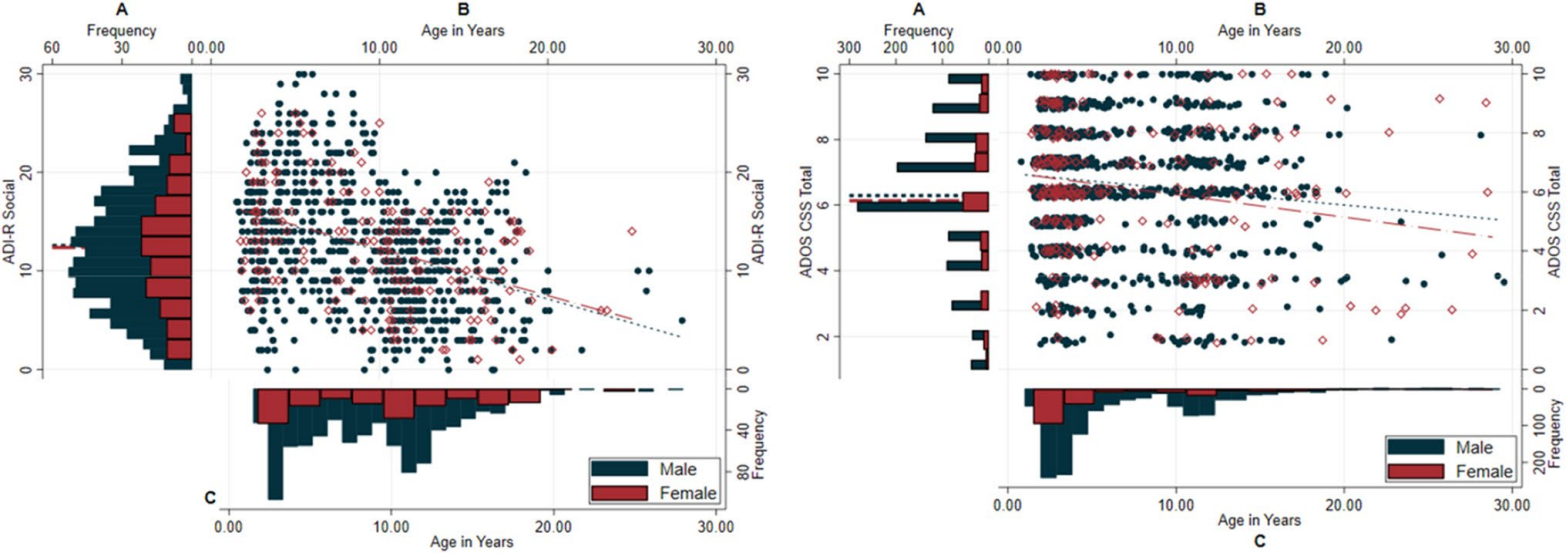
Whole sample—left panel: ADI-R Social domain current scores for males and for females, right panel: ADOS CSS Total scores for males and for females. **a** Distribution of scores for males (blue) and females (red), mean scores by sex presented in dashed lines; **b** Scatterplots of scores (Males: blue filled; Females: red hollow) with overlaid regression lines for males (blue dotted) and females (red dashed) separately; **c** Distribution of chronological age by sex. Note that for ease of presentation, only individuals aged up to 30 years are displayed here. (Color figure online)

Age-related analyses showed significant negative effects of age for ADI-R Social (*b* = − .41, *p* < .001, see Table 4; Fig. 2 left panel) and Communication domain current scores (*b* = − .23, *p* < .001), but not ADI-R RRB current scores (*b* = .01, *p* = .11). There were also significant negative effects of age for ADOS CSS Total (*b* = − .04, *p* = .002; see Fig. 2 right panel), but not ADOS CSS Social Affect (*b* = − .03, *p* = .03) and ADOS CSS Restricted and Repetitive Behaviours (RRB; *b* = − .01, *p* = .19). It is important to highlight that the vast majority of individuals with either ADOS CSS (97%) or ADI-R current scores (98%) fell within the 2–25 years’ age range, beyond which data for both measures was more limited (see Figure S1). This suggests that the significant differences in symptom scores as a function of age on these measures largely reflect differences across this particular age range rather than the entire age range of the sample.

To remove variance in the data due to differences between participants in cognitive abilities which might relate to scores on the ADOS or ADI-R, linear mixed-effects models were re-fitted using NVIQ as an additional predictor in a sub-sample of participants for whom NVIQ was available (see Table 5 for a summary of the results). After Bonferroni correction for multiple comparisons, sex-related analyses were approaching significance for ADI-R 4-to-5/ever scores on the RRB domain with males having higher scores than females (*M*_Male_ = 4.83; *SD*_Male_ = 3.4, *M*_Female_ = 4.47; *SD*_Female_ = 3.6, *x*^*2*^(1) = 5.07, *p* = .024, *d* = .21). All other comparisons between the sexes for ADOS CSS (Total, SA, RRB), ADI-R diagnostic scores (Social and Communication domain) and ADI-R current scores (Social, Communication, RRB) remained non-significant when controlling for NVIQ.

As with the previous analysis, a significant sex by age interaction for ADOS CSS RRB was not found to be robust to restricting the analysis to individuals younger than 25 years (accounting for a potential bias from limited data points and therefore wide confidence intervals in the older age groups). A significant main effect of age was retained for current scores on the ADI-R Social (*b* = − .29, *p* < .001) and Communication domain (*b* = − .19, *p* < .001), with older individuals having lower symptom scores than younger individuals, but not ADOS CSS total and CSS social affect.

## Discussion

This study investigated sex- and age-related differences in core ASD symptomatology as measured by the ADI-R and ADOS in a large and heterogeneous sample of 2684 individuals with ASD seen across 28 European clinical and research sites. Consistent with a meta-analysis of small-scale studies (Van Wijngaarden-Cremers et al. 2014) and findings from large-scale studies (Mandy et al. 2012; Szatmari et al. 2012; Frazier et al. 2014; Supekar and Menon 2015; Wilson et al. 2016; Charman et al. 2017), we found evidence of a lesser reported level of early childhood RRB on the ADI-R in females compared to males alongside comparable levels of reciprocal social interaction and communication difficulties at this age of presentation. In contrast to the present findings, some studies have also identified differences between girls and boys in early social symptoms on the ADI-R (Carter et al. 2007), but these findings are more limited and tended to report null effects when taking account of IQ (Banach et al. 2009; Lord et al. 1982).

While the overall patterns of results were maintained when non-verbal intellectual functioning was accounted for in the analyses, the significant finding of lower RRB in females relative to males dropped to a trend level after Bonferroni correcting for multiple comparisons. This makes the interesting proposition that non-verbal intellectual functioning can account and may attenuate some of the sex differences found in RRB in ASD. Alternatively, the lower significance level may also be related to a loss in statistical power due to analysing a smaller sample, which is supported by the observation that effect size estimates of sex comparisons were equivalent between the analyses. Note that regardless of whether age was accounted for in the analyses or not, the findings remained unchanged, suggesting that in this heterogeneous sample studied here, the presence/absence of sex differences in ASD severity was independent of age.

On current measures of RRB based on both caregiver interview and direct observation data, females showed as severe symptoms as males. This is at odds with some existing data demonstrating fewer current symptoms of RRB in females relative to males as measured by the ADOS (Bölte et al. 2011; Lai et al. 2011). One possible reason for differences in results may be the smaller sample size and narrower age range of the samples studied, i.e. adolescents (N = 56; Bölte et al. 2011) and adults-only (N = 83; Lai et al. 2011), compared to the much larger sample and broader age range reported in the present study from early childhood to adulthood. This may suggest that our sample composition obscured any age-dependent sex differences in RRB in adolescence and adulthood. While we did observe a significant sex by age interaction for RRB measured by the ADOS, supporting this suggestion, the results were not robust and likely the result of a small proportion of older male subjects with more severe RRB. Due to limited data points in this older age group, we were however unable to further test this hypothesis. It is important to point out that the present findings of equivalent RRB in females relative to males on the ADOS are consistent with other large-scale studies with similar age distributions (Charman et al. 2017; Frazier et al. 2014) and a recent study in adults with ASD (Wilson et al. 2016: sample N = 1244 adults with ASD; inter-quartile age range: 22–39 years). This potentially indicates that some of the previous findings of sex differences in current symptoms of RRB in adolescence and adulthood may have been sample- and/or study-specific. No sex differences relating to current social communication symptoms, as captured by the ADOS (CSS social affect) and ADI-R (social and communication domain scores), and overall ASD severity (ADOS CSS total) were observed. While this contradicts some reports of greater socio-communication difficulties on the ADOS in females (Carter et al. 2007; Hartley and Sikora 2009; Frazier et al. 2014), it is in line with others that identified no differences between the sexes (Holtmann et al. 2007; Bölte et al. 2011; Mandy et al. 2012; Reinhardt et al. 2015).

This study adds to the now growing literature that suggests that girls with ASD tend to show lesser levels of restricted interests, behaviours and stereotypes during the most ‘abnormal’ or ‘prototypic age’ of presentation, i.e. ever and 4-to-5-years, but exhibit a more similar autistic phenotype to boys in relation to social communication deficits both at younger and older ages. However, in the absence of longitudinal data in this study, conclusions about symptom trajectory or developmental changes should be considered with caution.

The current findings therefore indicate the presence of specific sex-related differences in the early developmental pattern of repetitive behaviours, routines and/or interests. What may be the factors that underlie this finding? One possibility could be etiologic protective factors, such that females have a higher liability threshold for expressing ASD symptoms compared to males, particularly for RRB (Szatmari et al. 2012). This is also consistent with behavioural genetic studies (Ronald et al. 2006; Robinson et al. 2016) highlighting the possibility for sex-and domain-specific protective factors (Constantino and Charman 2012, 2016). In the context of the skewed sex ratio in ASD towards a greater preponderance of males over females, a higher liability threshold for expressing RRB, particularly in higher-ability females with ASD, may contribute to the commonly reported widening of the sex ratio particularly at the intellectually able end of the spectrum.

Aside from a differential liability threshold, it may also be possible that higher-ability females are being under-identified as a result of displaying fewer RRB even if they present with considerable difficulties across other domains. This is in line with suggestions that clinicians are reluctant to consider a diagnosis of ASD without the presence of RRB (Mandy et al. 2012), and is reflected by the requirement for an ASD diagnosis in the DSM-5 for the presence of at least two significant indications of RRB, which is putting females at even greater risk of being unnoticed (Mandy et al. 2011). Alternatively, girls may simply exhibit ‘different’ rather than ‘fewer’ RRB than males which are therefore discounted during clinical and diagnostic assessments (Lai et al. 2015; see special issue in Autism; Mandy and Lai 2017). Clearly, future studies of the specific symptom patterns of females and how this relates to DSM-5 criteria are needed. Furthermore, early descriptions of ASD tended to be male-focussed (Kanner 1943) and diagnostic instruments including the ADI-R and ADOS were predominantly developed using male samples, leading potentially to a male-biased understanding of ASD and concomitant sex bias in the construct and item-structure of the instruments themselves. This may suggest that future revisions of these instruments require additional items to be included that are more characteristic of the female ASD phenotype. At least for the ADI-R, there is some evidence to suggest equivalent scale and item structure of the ASD phenotype in males and females (Duku et al. 2013; Frazier and Hardan 2017), but such evidence is missing for the ADOS. A future goal of research should therefore be continued exploration of the psychometric properties of these instruments (including establishing measurement equivalence across sexes) to evaluate the requirement for sex-specific norms (Constantino and Charman 2016; Lai et al. 2015). Future studies will also benefit from investigating sex differences using instruments that might be more sensitive to potential sex differences in presentation of ASD characteristics also outside of the clinical arena, such as the SRS-2 (Constantino 2012), a parent, teacher, spouse, and/or self-report questionnaire measure of autistic—like traits (Frazier et al. 2014; Howe et al. 2015; Charman et al. 2017; Ratto et al. 2017), compared to the ‘gold-standard’ diagnostic instruments the ADI-R and ADOS used in the current study.

Another possibility for the current results is that rater reports may have influenced the findings. Mothers are typically the primary source of information during diagnostic assessments and sex differences reported on the ADI-R may be a function of parents reporting symptoms differently for girls and boys. In the current study however, we were unable to further assess these possibilities. Lastly, the current results may also potentially reflect sex differences in RRB in early typical development. However, while some studies have found boys to score higher than girls on ratings of repetitive behaviours and preoccupations with restricted patterns of interest, but not repetitive movements, sensory interest, or rigidity (Leekam et al. 2007), others have not demonstrated sex differences in RRB in early development (Evans et al. 1997; Øien et al. 2017).

Age-related analyses revealed lower current social and communication symptoms with age as measured by the ADI-R, both with and without covarying for NVIQ, with older subjects reporting lower symptom scores than younger subjects. Since the majority of participants fell within the 2–25 years’ age range, beyond which data was more limited, the significant differences in symptom scores as a function of age largely reflected differences across this particular age range rather than the entire sample. ADOS CSS total and CSS social affect displayed a similar albeit attenuated effect of a negative relationship between symptom scores and age, which however disappeared when non-verbal intellectual functioning was accounted for in the analyses. These results broadly support a range of studies showing reduced ASD symptoms with increasing age, including those studies that tracked samples longitudinally since childhood (Billstedt et al. 2007; Howlin et al. 2013; Shattuck et al. 2007). Larger cross-sectional samples that have also reported differences in symptomatology with age are rare, but those that did, did not find significant age differences on the ADOS when IQ was included in the model (e.g. N = 325, Mandy et al. 2012; N = 437; Charman et al. 2017). Given the cross-sectional nature of the data, it is not clear if the age-related differences observed reflect true effects or are due to sampling differences between datasets that recruited participants across different ages.

### Limitations

Although the total sample size of the current study was large, the sample consisted of individual datasets pooled across many different sites that were not fully matched for assessment methodologies, diagnostic procedures and ascertainment strategies. Also, samples were derived across different research programmes with different purposes (e.g. early screening studies, intervention programs, high-risk sibling studies, genetic and imaging studies), and differed in respect to the distribution and range of ASD symptom severity, age and intellectual functioning. However, unfortunately, the individual sample sizes for each dataset were too small to allow for any additional meaningful comparisons within individual datasets.

It is also important to acknowledge that for data relating to the ADOS, participants were not equally distributed across the different modules, with the majority of subjects completing Module 1 designed for individuals who are preverbal or who use single words to communicate. This somewhat limits the conclusions drawn in relation to age-related trends in the ADOS data.

## Conclusions

Pooling datasets across European clinical and research sites allowed us to analyse sex and age-related differences in ADOS and ADI-R in one of the largest ASD samples studied to-date. The size and heterogeneous nature of the datasets collected, both in relation to age, IQ and cultural factors, circumvented previous limitations of low statistical power due to small samples, narrow age and IQ ranges, which may, in part, explain some of the inconsistencies found in earlier studies. We identified some phenotypic differences between males and females, particularly in relation to early childhood symptoms of RRB, but found little evidence for sex differences in social communication deficits both at younger and older ages. We also observed lower social-communicative symptoms in older compared to younger individuals with ASD, consistent with previous longitudinal studies. A better understanding of sex differences in ASD symptom presentation is motivated by the need to improve recognition and diagnosis in females to facilitate support that can follow from an ASD diagnosis in the form of early interventions and targeted health care and educational programs for the child and family. In addition, it may help to elucidate important basic science questions to better understand the neurobiological and/or developmental mechanisms that potentially underlie some of the differences in ASD symptom expression.

## Electronic Supplementary material

Below is the link to the electronic supplementary material.

Supplementary material 1 (DOCX 271 KB)
