# Supplementary material for: Evaluating Sex and Age Differences in ADI-R and ADOS Scores in a Large European Multi-site Sample of Individuals with Autism Spectrum Disorder
Source: J Autism Dev Disord. 2018 Feb 21;48(7):2490–505. doi: 10.1007/s10803-018-3510-4 (PMC5996001; doi:10.1007/s10803-018-3510-4)
Supplement: Supplementary file 1 — Supplementary material 1 (DOCX 271 KB) [file 10803_2018_3510_MOESM1_ESM.docx]

**Article title**: Evaluating sex and age differences in ADI-R and ADOS scores in a large European multi-site sample of individuals with Autism Spectrum Disorder
**Journal name:** Journal of Autism and Developmental Disorders
**Author names**: Tillmann, J., Ashwood, K., Absoud, M., Bölte, S., Bonnet-Brilhault, F., Buitelaar, J. K., Calderoni, S., Calvo, R., Canal-Bedia, R., Canitano, R., De Bildt, A., Gomot, M., Hoekstra, P. J., Kaale, A., McConachie, H., Murphy, D. G., Narzisi, A., Oosterling, I., Pejovic-Milovancevic, M., Persico, A.M., Puig, O., Roeyers, H., Rommelse, N. , Sacco, R., Scandurra, V., Stanfield, A. C., Zander, E., Charman, T.
E-mail address of the corresponding author: julian.tillmann@kcl.ac.uk

Supplementary Materials

*Sex differences in chronological age and intellectual functioning by dataset*

There were no significant age differences between males and females for ADOS-only datasets (*M*_Male_ = 7.3, *SD*_Male_ = 7.4; *M*_Female_ *=* 7.7, *SD*_Female_ = 7.3, *x^2^*(1) = 1.42, *p* = .234, *d* = .03), ADI-R 4-5 ever/diagnostic datasets (*M*_Male_ = 11.0, *SD*_Male_ = 8.7; *M*_Female_ *=* 12.2, *SD*_Female_ = 9.5, *x^2^*(1) = 0.01, *p* = .925, *d* = .01) and ADI-R current datasets (*M*_Male_ = 9.1, *SD*_Male_ = 5.5; *M*_Female_ *=* 10.3, *SD*_Female_ = 6.9, *x^2^*(1) = 2.95, *p* = .09, *d* = .05).

The pattern of significantly higher non-verbal intellectual abilities (NVIQ) in males relative to females in the whole sample was also observed when splitting the total sample into ADOS-only datasets (NVIQ: *M*_Male_ = 74.4, *SD*_Male_ = 26.0; *M*_Female_ *=* 69.9, *SD*_Female_ = 27.9, *x^2^*(1) = 7.81, *p* = .005, *d* = .26), ADI-R 4-to-5 diagnostic/ever datasets (NVIQ: *M*_Male_ = 81., *SD*_Male_ = 26.9; *M*_Female_ *=* 75.7, *SD*_Female_ = 28.4, *x^2^*(1) = 14.56, *p* = .0001, *d* = .32) and ADI-R current datasets (NVIQ: *M*_Male_ = 82.1, *SD*_Male_ = 27.4; *M*_Female_ *=* 75.8, *SD*_Female_ = 29.8, *x^2^*(1) = 13.02, *p* = .0003, *d* = .37).

*Statistical analysis for combined ADOS and ADI-R datasets*

Linear mixed-effects models were fit using dataset as a random effect and age, NVIQ and sex as continuous/categorical predictors and run for participants with both ADI-R and ADOS data only (*N* = 678). Supplementary Table 2 shows a summary of the regression models including standard errors, t-statistics and confidence intervals for slope coefficients (age and NVIQ) and chi-square coefficients and p-value for categorical predictor variables (sex).

Increased chronological age was significantly associated with lower current symptom scores on the ADI-R Social domain (*b* = -.22, *p* < .001, see Figure S1 left panel) and ADI-R Communication (*b* = -.09, *p* = .002, see Figure S1 right panel), but not ADI-R RRB current scores (*p* > .6). Age was not significantly associated with Calibrated Severity Scores (CSS) on the ADOS. On the ADI-R RRB domain, sex differences were approaching significance with males having higher 4-5 diagnostic/ever scores than females (*x^2^*(1) = 4.35, *p* = .037, *d* = .25, Bonferroni-corrected *p*-value). Higher intellectual functioning (Non-verbal IQ score) was significantly associated with lower scores on all clinical measures (ADOS, ADI-R 4-5 diagnostic/ever scores, ADI-R current scores; all *p* <. 002).

Table S1 Summary statistics of ADI-R and ADOS scores for females and males

|  | **Females** | | |  | **Males** | | |
| --- | --- | --- | --- | --- | --- | --- | --- |
| Variable | *N* | Mean (95% *CI*) | *SD* (95% *CI*) |  | *N* | Mean (95% *CI*) | *SD* (95% *CI*) |
| **ADI-R – 4-5 ever/item scores** |  |  |  |  |  |  |  |
| Social | 376 | 16.69 [15.96,17.41] | 7.14 [6.66,7.69] |  | 1763 | 17.55 [17.24,17.87] | 6.78 [6.56,7.01] |
| Communication | 365 | 11.73 [11.23,12.22] | 4.83 [4.51,5.21] |  | 1746 | 12.58 [12.35,12.81] | 4.93 [4.77,5.10] |
| Restricted and  Repetitive Behaviours^b^ | 367 | 4.39 [4.05,4.72] | 3.28 [3.06,3.54] |  | 1730 | 5.05 [4.90,5.19] | 3.16 [3.06,3.27] |
|  |  |  |  |  |  |  |  |
| **ADI-R - Current scores** |  |  |  |  |  |  |  |
| Social | 169 | 12.14 [11.23,13.04] | 6.02 [5.44,6.75] |  | 861 | 12.52 [12.11,12.94] | 6.19 [5.91,6.50] |
| Communication | 158 | 8.99 [8.36,9.62] | 4.04 [3.63,4.54] |  | 844 | 9.58[9.30,9.85] | 4.06 [3.87,4.26] |
| Restricted and  Repetitive Behaviours^b^ | 170 | 3.51 [3.16,3.86] | 2.33 [2.11,2.61] |  | 867 | 4.12 [3.95,4.29] | 2.51 [2.39,2.63] |
|  |  |  |  |  |  |  |  |
| **ADOS Calibrated Severity Scores** |  |  |  |  |  |  |  |
| Total | 233 | 6.09 [5.79,6.38] | 2.32[2.13,2.56] |  | 1187 | 6.26 [6.13,6.39] | 2.25 [2.16,2.35] |
| Social Affect | 233 | 6.49 [6.19,6.79] | 2.34 [2.15,2.58] |  | 1187 | 6.55 [6.42,6.68] | 2.24 [2.15,2.33] |
| Restricted and  Repetitive Behaviours^b^ | 233 | 5.69 [5.36,6.03] | 2.61 [2.39,2.87] |  | 1187 | 6.20 [6.06,6.35] | 2.50 [2.40,2.60] |

Note: *SD* = Standard Deviation, 95% *CI* = 95% Confidence Interval of mean/standard deviation; ADI-R = Autism Diagnostic Interview – Revised; ADOS = Autism Diagnostic Observation Schedule

Table S2 Sub-sample with both ADI-R and ADOS data - effect of age, sex and IQ

|  | **Chronological age** | | | |  | **Intellectual functioning** | | | |  | **Sex** | | |  | **Sex by age interaction** | | | |
| --- | --- | --- | --- | --- | --- | --- | --- | --- | --- | --- | --- | --- | --- | --- | --- | --- | --- | --- |
| Variable | *b*  *SE(b)* | *t* | *p-value* | 95% *CI* |  | *b*  *SE(b)* | *t* | *p-value* | 95% *CI* |  | *x^2^* | *p-value* | *d* |  | *b*  *SE(b)* | *t* | *p*-value | 95% *CI* |
| **ADI-R – 4-5 ever/item scores**^a^ | | | | | | | | | | | | | | | | | | |
| Social |  |  |  |  |  | -.07  (.01) | 8.19 | < .001 | [-.09, -.06] |  | 0.32 | .569 | .05 |  |  |  |  |  |
| Communication |  |  |  |  |  | -.02  (.01) | 3.26 | .001 | [-.04, -.01] |  | 1.35 | .245 | .12 |  |  |  |  |  |
| Restricted and  Repetitive Behaviours^b^ |  |  |  |  |  | -.01  (.01) | 3.14 | .002 | [-.01, -.01] |  | 4.35 | .037 | .25 |  |  |  |  |  |
| **ADI-R - Current scores**^c^ | | | | | | | | | | | | | | | | | | |
| Social | -.22  (.05) | 4.08 | < .001 | [-.33, -.12] |  | -.07  (.01) | 7.58 | < .001 | [-.09, -.05] |  | 1.01 | .315 | .06 |  | -.08  (.08) | 1.06 | .289 | [-.24, .07] |
| Communication | -.09  (.03) | 3.10 | .002 | [-.15, -.03] |  | -.03  (.01) | 5.34 | < .001 | [-.05, -.02] |  | 0.15 | .699 | .10 |  | -.07  (.06) | 1.28 | .202 | [-.19, .04] |
| Restricted and  Repetitive Behaviours^b^ | .01  (.01) | 0.39 | .698 | [-.01, .02] |  | -.01  (.01) | 3.98 | < .001 | [-.01, -.01] |  | 3.21 | .073 | .42 |  | -.01  (.01) | 0.34 | .734 | [-.02, .02] |
| **ADOS Calibrated Severity Scores**^d^ | | | | | | | | | | | | | | | | | | |
| Total | .01  (.02) | 0.01 | .992 | [-.04, .04] |  | -.03  (.01) | 9.57 | < .001 | [-.04, -.03] |  | 0.88 | .349 | .19 |  | -.01  (.03) | 0.44 | .667 | [-.07, .05] |
| Social Affect | .01  (.02) | 0.33 | .740 | [-.03, .05] |  | -.03  (.01) | 8.93 | < .001 | [-.04, -.02] |  | 1.91 | .166 | .19 |  | .01  (.03) | 0.15 | .884 | [-.06, .06] |
| Restricted and  Repetitive Behaviours^b^ | -.01  (.01) | 0.02 | .982 | [-.01, .01] |  | -.01  (.01) | 8.48 | < .001 | [-.01, -.01] |  | 0.03 | .872 | .13 |  | -.01  (.01) | 0.99 | .321 | [-.03, .01] |

Note: *b* = regression coefficient, *SE(b)* = standard error of regression coefficient, *t* = t-statistic, 95% *CI* = 95% Confidence Interval of regression coefficient
ADI-R = Autism Diagnostic Interview – Revised; ADOS = Autism Diagnostic Observation Schedule

^a^ ADI-R 4-to-5 diagnostic/ever scores analyses: *N*=678 participants included; ^b^ log-transformed scores; ^c^ ADI-R current scores analyses: *N*=462 participants included; ^d^ADOS analyses: *N*=678 participants included


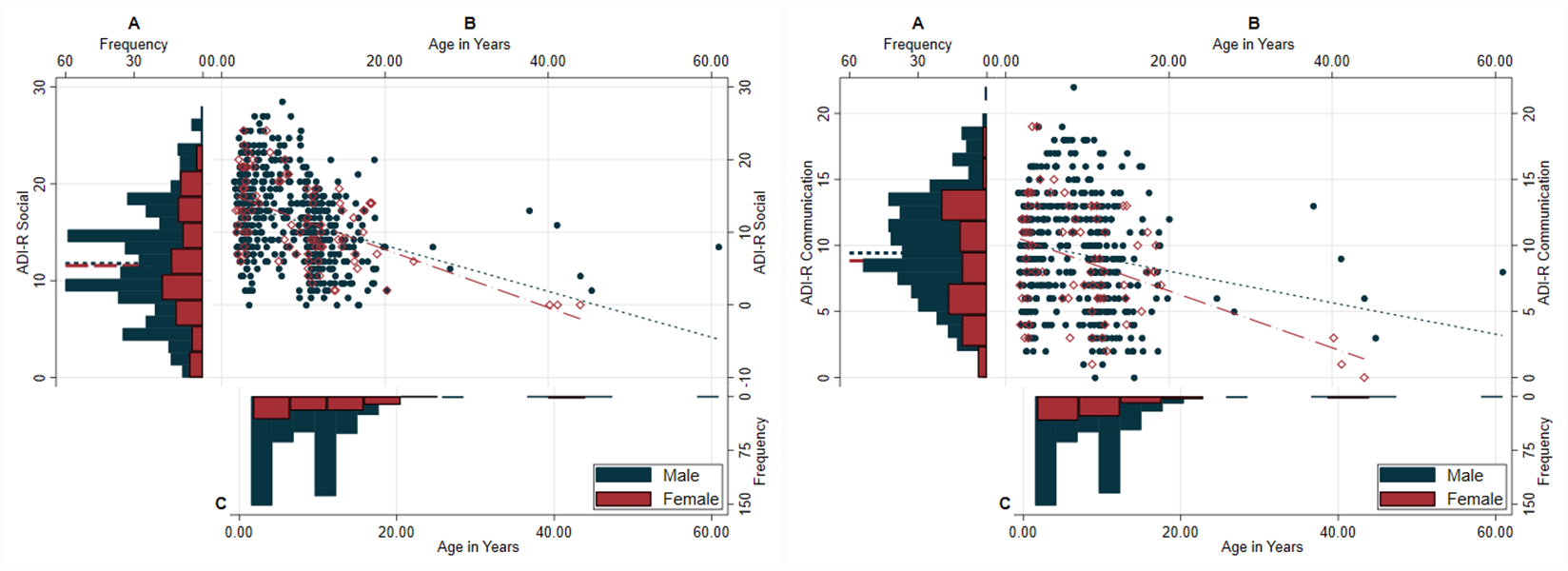


**Fig. S1** Whole sample - Left panel: ADI-R Social domain current scores for males and for females, right panel: ADI-R Communication domain current scores for males and for females. (A) Distribution of scores for males (blue) and females (red), mean scores by sex presented in dashed lines; (B) Scatterplots of scores (Males: blue filled; Females: red hollow) with overlaid regression lines for males (blue dotted) and females (red dashed) separately; (C) Distribution of chronological age by sex.
